# Epidemics and Frequent Recombination within Species in Outbreaks of Human Enterovirus B-Associated Hand, Foot and Mouth Disease in Shandong China in 2010 and 2011

**DOI:** 10.1371/journal.pone.0067157

**Published:** 2013-06-19

**Authors:** Ting Zhang, Jiang Du, Ying Xue, Haoxiang Su, Fan Yang, Qi Jin

**Affiliations:** MOH Key Laboratory of Systems Biology of Pathogens, Institute of Pathogen Biology, Chinese Academy Medical Sciences & Peking Union Medical College, Beijing, China; University of Illinois at Chicago, United States of America

## Abstract

The epidemiology and molecular characteristics of human enterovirus B (HEV-B) associated with hand, foot and mouth disease (HFMD) outbreaks in China are not well known. In the present study, we tested 201 HEV isolates from 233 clinical specimens from patients with severe HFMD during 2010–2011 in Linyi, Shandong, China. Of the 201 isolates, 189 were fully typed and 18 corresponded to HEV-B species (six serotypes CVA9, CVB1, CVB4, Echo 6, Echo 25 and Echo 30) using sensitive semi-nested polymerase chain reaction analysis of VP1 gene sequences. Phylogenetic analysis based on the VP1 region showed that eight E30SD belonged to a novel sub-genogroup D2; E25SD belonged to a novel sub-genogroup D6; E6SD belonged to sub-lineage C6 and five CVB1SD belonged to subgroup 4C; and B4SD belonged sub-lineage D2. The full viral genomes of the CVB1SD, E6SD, E25SD and E30SD isolates were sequenced. Analysis of phylogenetic and similarity plots indicated that E25SD recombined with E25-HN-2, E30FDJS03 and E4AUS250 at noncontiguous P2A–P3D regions, while E30SD, E30FDJ03, E25-HN-2 and E9 DM had shared sequences in discrete regions of P2 and P3. Both E6SD and B1SD shared sequences with E1-HN, B4/GX/10, B5-HN, and A9-Alberta in contiguous regions of most of P2 and P3. Genetic algorithm recombination detection analysis further confirmed the existence of multiple potential recombination points. In conclusion, analysis of the complete genomes of E25SD, E30SD, CVB1SD and E6SD isolated from HFMD patients revealed that they formed novel subgenogroup. Given the prevalence and recombination of these viruses in outbreaks of HFMD, persistent surveillance of HFMD-associated HEV-B pathogens is required to predict potential emerging viruses and related disease outbreaks.

## Introduction

EVs (genus *Enterovirus*, family *Picornaviridae*) contain positive-sense single- stranded RNA genomes and are the causative agents of various diseases, including herpangina, hand-foot-mouth disease (HFMD), meningitis, and nonspecific febrile illnesses [1]. HEVs are evolutionarily classified into 4 species (HEV-A to HEV-D) based on the molecular and antigenic properties of the viruses. The 5′-untranslated regions (5′UTR), capsid protein (P1 region) and non-structural protein (NSP, P1 and P2 regions) of the HEV-B genome evolve virtually independently, and frequently recombine to give rise to new virus variants [2]. HEV-B thus comprises 60 conventional serotypes, including Cox B1-6, A9, Echo 1–7, 9, 11–21, 24–27, and 29–33, and EV-B 69,73–75, 77–78, 93,97–98,100–101,106–107, 110 and Simian agent 5 (SA5), which are closely related to simian enteroviruses [3]. CVB1 and E30 are usually associated with severe neonatal illnesses and aseptic meningitis outbreaks and are the most commonly reported serotype in the United States [4], [5]. E30 is also receiving increasing attention in China in relation to outbreaks of aseptic meningitis in Jiangsu and the neighboring provinces of Zhejiang and Shandong [6], [7], [8]. E6 and E25 isolates are also both epidemic echovirus types capable of causing sporadic infections and outbreaks of aseptic meningitis [9], [10]. Four strains of E25 have been isolated from cerebrospinal fluid samples from patients in China's Henan Province [11].

HFMD usually affects children and has emerged as a significant public health issue in China in recent years. In 2010 and 2011, a total of 3,394,375 cases of HFMD were recorded in Mainland China, accounting for 1,414 deaths, according to the Ministry of Health of the People's Republic of China (http://www.moh.gov.cn/mohjbyfkzj/s3578/201202/54106.shtml). EV-71 and CV-A16 are the major etiological agents of HFMD [12], [13], [14], but other HEV-A serotypes, CV-A2-8, CV-A9-12, have also been associated with both sporadic infections and outbreaks of HFMD [15], [16]. In recent years, some HEV-B serotypes have also been detected in HFMD. A distinct new serotype CVB5 was first reported in 2009 when it was involved in an outbreak of EV-71-associated neurologic HFMD in Linyi City [17]. In addition, other HEV-B serotypes, E1, E6, E11, E13, E25 and E30, were also isolated under the HFMD surveillance network during a 6-month period in 2010, in Henan province, China[18]. E30- and EV71-associated HFMD outbreaks were also detected in Guangxi, Southern China in 2011 [19]. However, the epidemiology and molecular characteristics of the HEV-B species associated with HFMD outbreaks in China are still not well known. Moreover, 10–30% isolates from HFMD samples were unable to be characterized in many reports.

In this study, we investigated the presence of enteroviruses in throat swabs from >233 patients in Linyi City in 2010 and 2011, and fully typed 187 isolates from 201 HEVs based on VP1 gene analyses. We present four full-length genomic sequences of the modern HEV-B strains, CVB1SD2011CHN (B1SD), E6SD2011CHN (E6SD), E25SD2010CHN (E25SD) and E30SD2010CHN (E30SD), which represent HEV-B serotypes CVB1, Echo6, Echo25 and Echo30, respectively. We compared the four strains with other related viruses in terms of their partial 5′ untranslated regions (UTRs) and P1/P2/P3 regions. Understanding the hereditary and molecular epidemiology of HEV-B will have important implications on epidemiological investigations and the prevention and control of HFMD.

## Results

### Enterovirus detection and molecular typing

**Figure 1:**
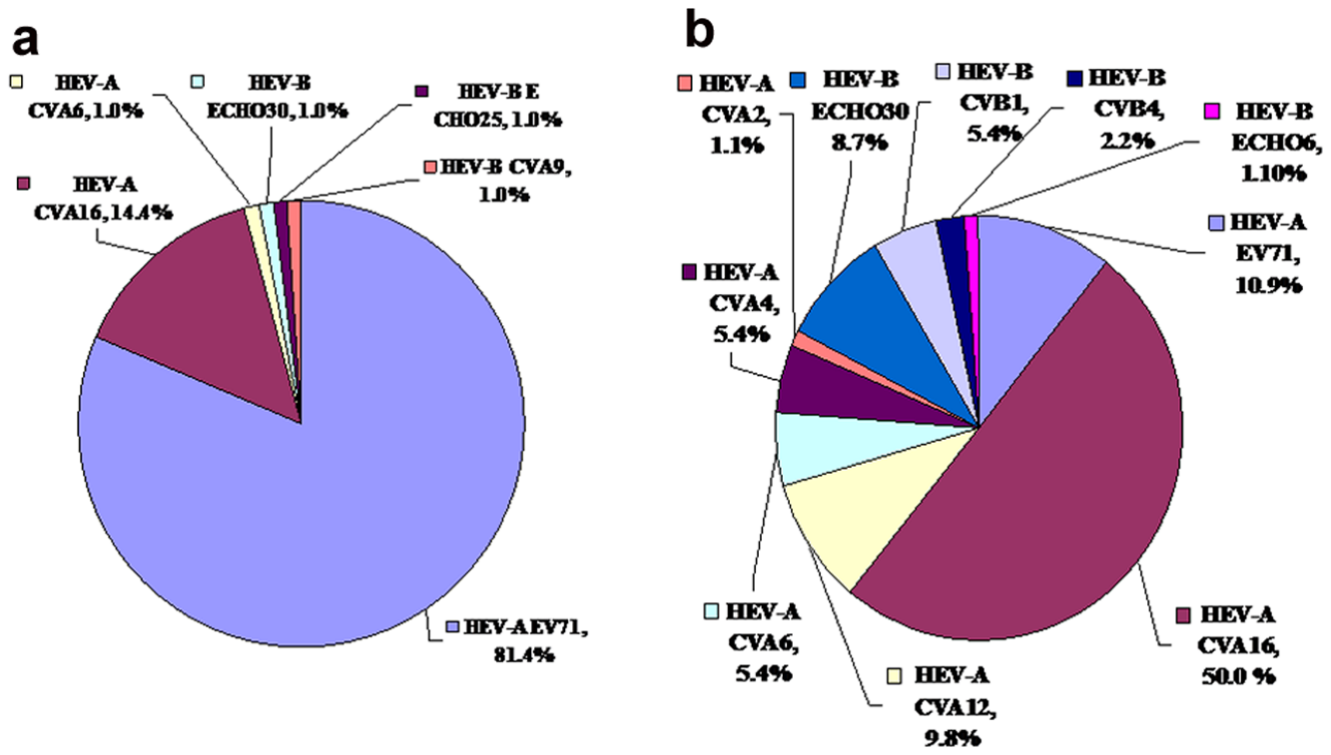
Distribution of human echovirus serotypes from hospitalized HFMD cases in Linyi, China, 2010–211. Percentage of each serotype among total isolates for each year. (a) HEV in 2010; (b) HEV in 2011. Echo, Echovirus; CAV, Coxsackievirus A; CBV, Coxsackievirus B.

A total of 201 enterovirus isolates from 233 samples from patients with severe HFMD were tested in 2010 and 2011. Enterovirus serotypes were confirmed using sensitive semi-nested PCR with cell culture that generated 372-bp amplicons, corresponding to the VP1 region. Of the 201 tested HEVs, 189 could be fully typed. Of the 97 typed in 2010 (Figure 1a), 94 (97%) were HEV-A (six different serotypes), three (3%) were HEV-B (three different serotypes, E25, E30 and A9), and 81.4% and 14.4% corresponded to the EV71 and CA16 serotypes, respectively. EV71 and CA16, with positivity rates of 10.9% (10/92) and 50.0% (46/92), respectively, remained the major pathogens of HFMD in 2011 (Figure 1b). However, among the 92 isolates typed in 2011, the percentage of HEV-A serotypes fell to 82.6% (76/92), while HEV-B serotypes increased to 17.4% (16/92), and 50% (8/16) of the HEV-B serotypes were Echo 30. Echo 30 was detected in two consecutive years. Five CVB1, two CVB4 and 1 Echo6 strains were detected for the first time in 2011, while Echo 25 and CVA9 were only detected in 2010, during an outbreak of HFMD in LinYi City, Shangdong province.

### Epidemiological features of enterovirus in Shangdong LinYi

The temporal distribution of the epidemic enterovirus in LinYi City was seasonal, with most cases occurring during the summer from June to September. In 2010–2011, 167 (88.4%) of 189 cases involved individuals younger than 4 years old, and 22 cases (11.6%) involved individuals between 4 and 8 years old. The highest rates of enterovirus-positive samples were from patients younger than 2 years old (75.2% in 2010 and 61.0% in 2011). Of the total 189 isolates, 128 were from males and 61 were from females, giving a male-to-female ratio of approximately 2.15∶1. The major clinical symptoms in hospitalized patients with enterovirus-related HFMD were vesicles on the hands, feet, buccal mucosa and tongue, accompanied by fever, vomiting, poor spirit, bilateral sounds of heavy breathing and instability when walking. In addition, at least 50% of EV71-associated HFMD patients also had lung infections, and Cox-As show herpangina symptoms. HFMD patients with HEV-B infection complicated by aseptic meningitis were mostly infected with Echo30 (70%).

### Molecular epidemiology of CVB1SD, CVB4SD, E6SD, E25SD and E30SD isolates based on VP1

**Figure 2:**
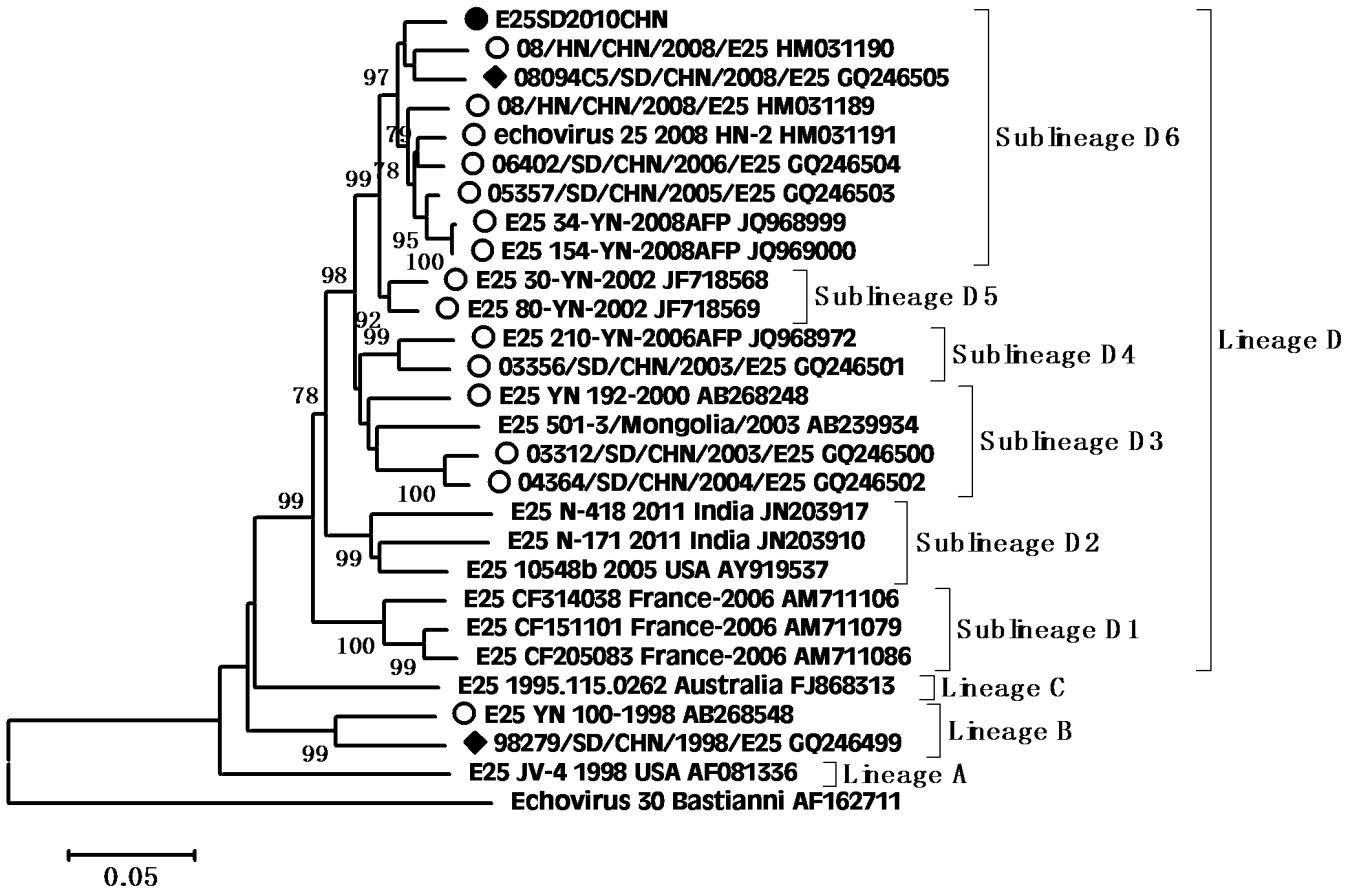
Phylogeny of E25 based on 875 nt of the VP1 gene generated by the neighbor-joining algorithm implemented in MEGA (version 5.0) using the Kimura two-parameter substitution model and 1000 bootstrap pseudo-replicates. •Strains isolated in this investigation. □Strains isolated from Shandong. ○Strains isolated from other provinces of China.

**Figure 3:**
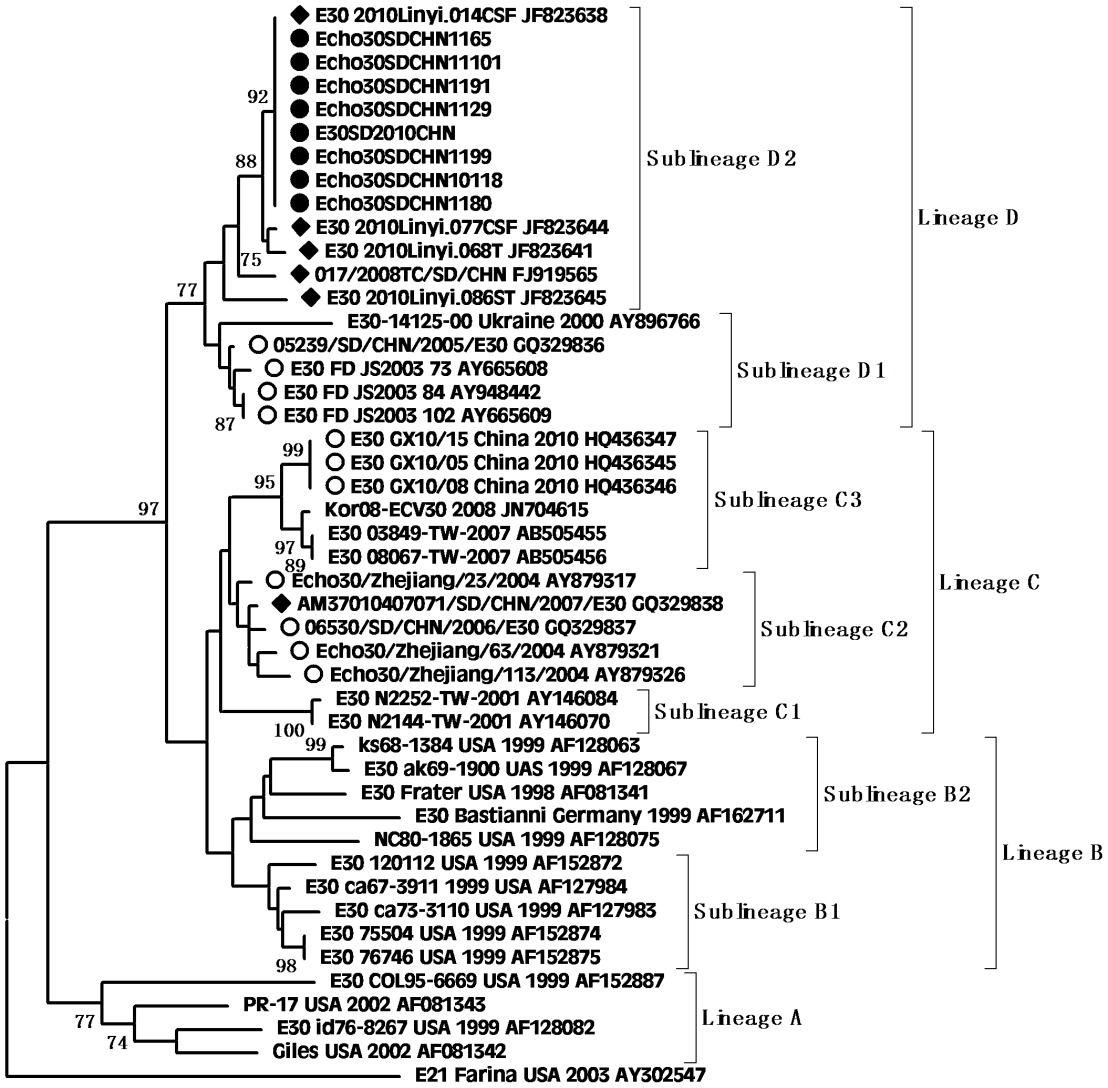
Phylogeny of E30 based on 875 nt of the VP1 gene generated by the neighbor-joining algorithm implemented in MEGA (version 5.0) using the Kimura two-parameter substitution model and 1000 bootstrap pseudo-replicates. •Strains isolated in this investigation. ♦Strains isolated from Shandong. ○Strains isolated from other provinces of China.

**Figure 4:**
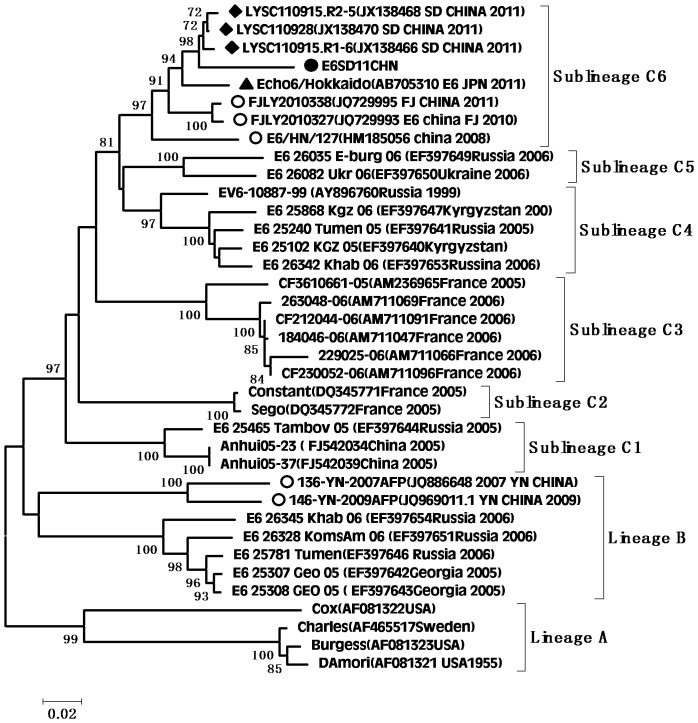
Phylogeny of E6 based on 875 nucleotides of the VP1 gene generated by the neighbor-joining algorithm implemented in MEGA (version 5.0) software using the Kimura 2-parameter substitution model and 1000 bootstrap pseudo-replicates. •Strains isolated in this investigation. ♦Strains isolated from Shandong. ○Strains isolated from other provinces of China.

**Figure 5:**
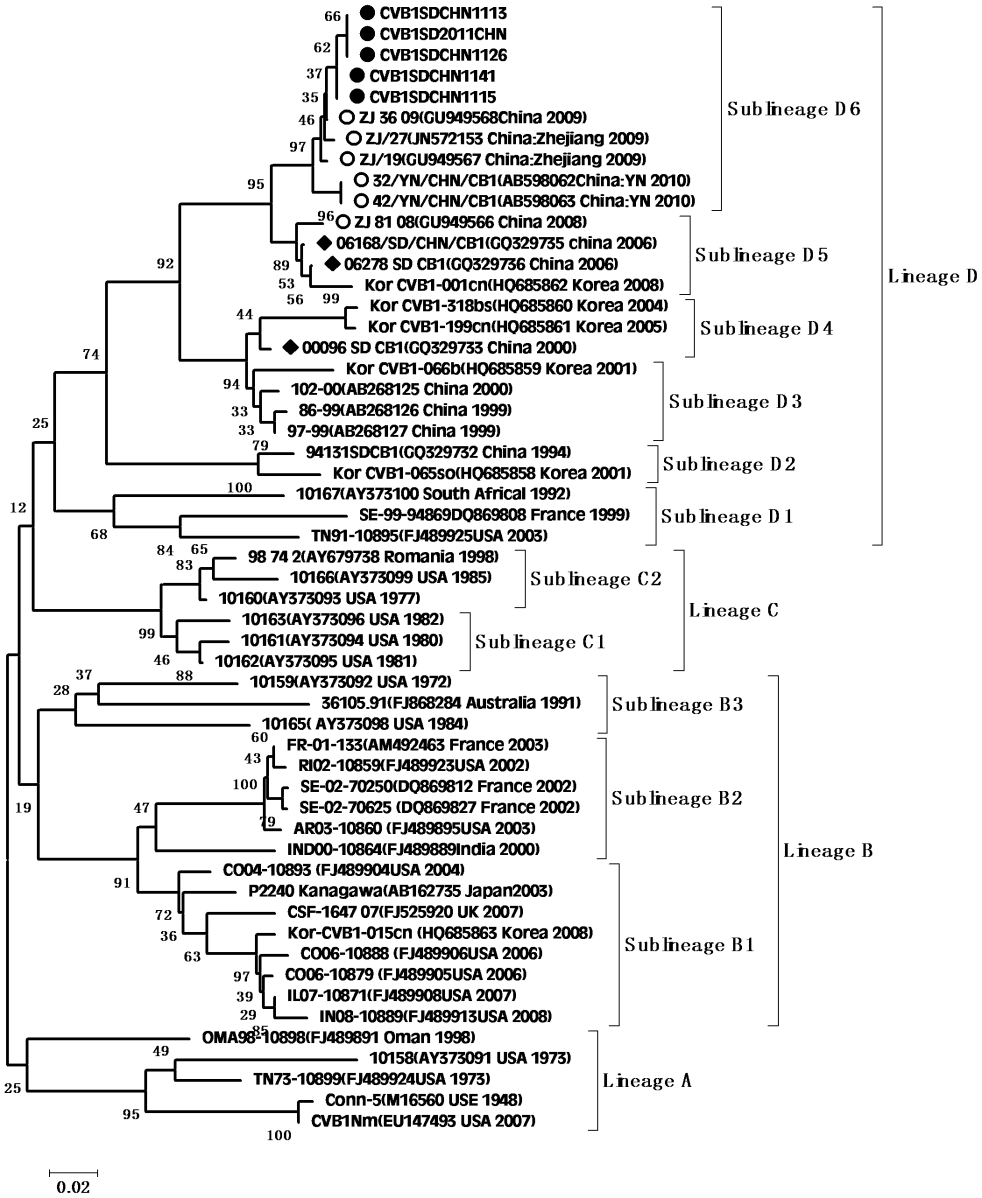
Phylogeny of CVB1 based on 475 nucleotides of the VP1 gene generated by the neighbor-joining algorithm implemented in MEGA (version 5.0) software using the Kimura 2-parameter substitution model and 1000 bootstrap pseudo-replicates. •Strains isolated in this investigation. ♦Strains isolated from Shandong. ○Strains isolated from other provinces of China.

**Figure 6:**
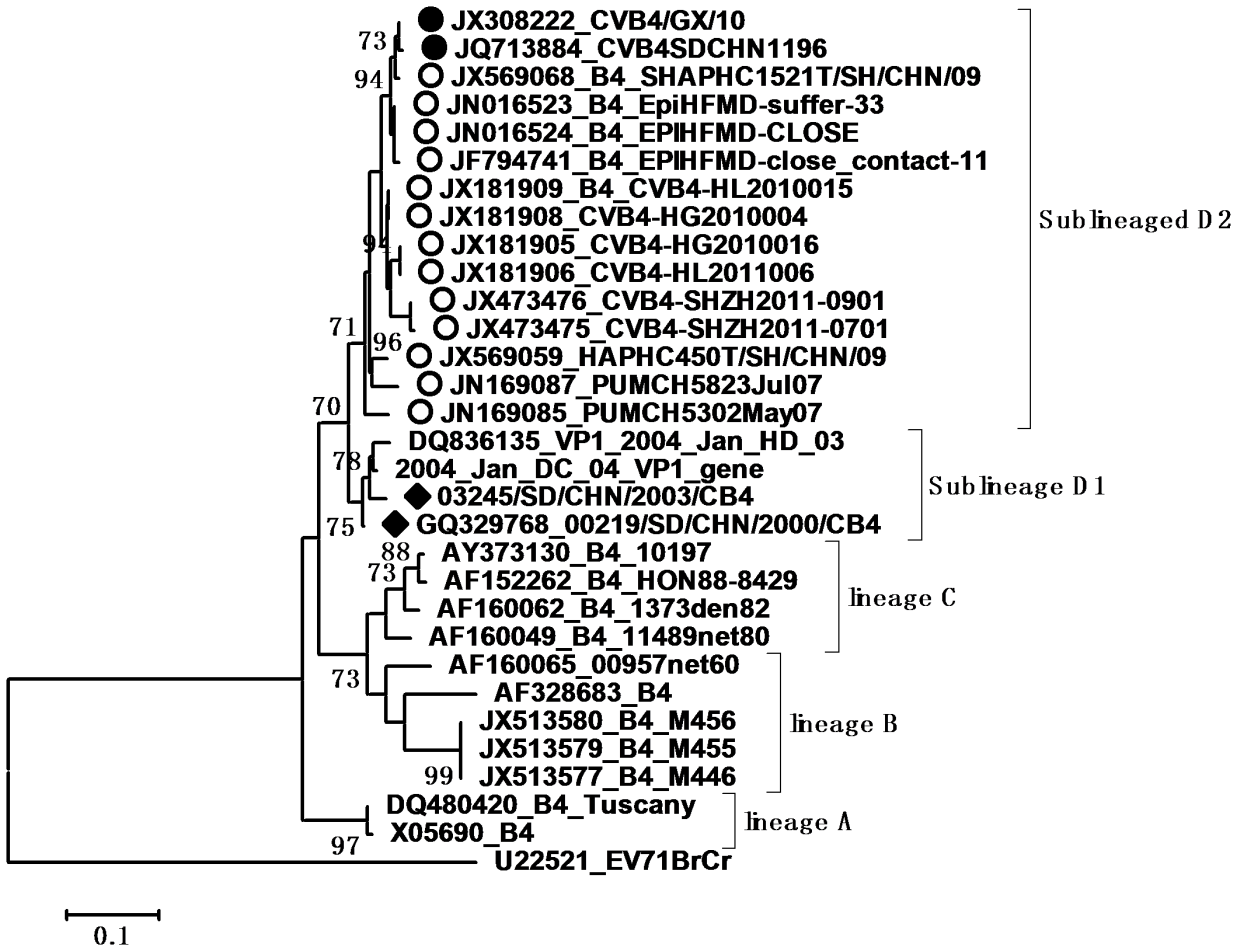
Phylogeny of CVB4 based on 475 nt of the VP1 gene generated by the neighbor-joining algorithm implemented in MEGA (version 5.0) using the Kimura two-parameter substitution model and 1000 bootstrap pseudo-replicates. •Strains isolated in this investigation. ♦Strains isolated from Shandong. ○Strains isolated from other provinces of China.

According to the molecular epidemiological study, genotypes were defined as clusters of related strains with >85% nt sequence identity in the VP1 region [20]. Individual phylogenetic dendrograms for E6, CVB1, CVB4, E25SD and E30SD were drawn based on VP1 sequences from all representative strains responsible for international or domestic epidemics during the past decade. There were four E25 genetic groups, designated A–D (subgroups D1–D6) [11].The E25SD10 sequence detected in the present study belonged to the novel subgenogroup D6 (Figure 2). Four genetic groups were classified for the E30 strains, designated A–D (subgroups D1–D2) [7], [21]. The Ukraine and E30 JS2003 strains belonged to subgroup D1, while the E30SD strains isolated in this study and E30Linyi (the latest strains available in GenBank) were categorized as novel subgenogroup D2 with nt sequence identities ranging from 95.6–98.9%, and amino acid identities ranging from 98.9–100% (Figure 3), which have not been reported previously. The E30 TW, Zhejiang, and GX strains belonged to group C. There were three Echo6 genetic groups designated A–C (subgroups C1–6), and four CVB1 genetic groups designated A–D (subgenogroups D1–6), which had been previously identified [4], [9]. Based on this classification, the E6SD sequence detected in the present study belonged to subgroup C6 (Figure 4) and five B1SD sequences belonged to the sublineage D6 (Figure 5). Finally, CVB4 was classified into three genetic groups A–C (subgroup C1–2), and two CVB4 sequences formed a cluster in the C2 sublineage (Figure 6). In addition, phylogenetic dendrograms of the EV71 and CA16 strains indicated that E71 and CA16 formed new subgenogroups C4a and C3c, respectively, as shown in Figures S1 and S2.

### Genomic features

We obtained complete genome sequences for the four HEV-B strains, CVB1SD, E6SD, E25SD and E30SD, with lengths of 7,368, 7,395, 7,422 and 7,427 nt, respectively. Their genomic features were analyzed by multiple comparisons with GenBank sequences of other HEV-B enteroviruses that were previously reported as prevalent in China, including 21 prototype strains and 20 modern strains. Pairwise comparisons for CVB1, E6, E25SD and E30SD with the 876-bp VP1 region of other HEV-B strains revealed that the viruses were most similar to prototypes Conn-5B1, Damori E6, E25-JV4, and E30 Bastianni with nt identities of 75.2, 77.2, 81.7 and 82.3%, and amino acid identities of 88.9, 93.6, 92.7 and 95.8%, respectively. VP1 analyses identified the four isolated strains as CVB1, Echo6, Echo 25 and Echo30, respectively. The nt identities of all HEV-B strains in the NSP regions P2 and P3 ranged from 69.1–97%, and the amino acid sequence identities of all HEV-B strains were similar, and ranged from 94.6–98%.

### Phylogenetic and recombination analysis of the four HEV-B strains and other HEV-B genomes

**Figure 7:**
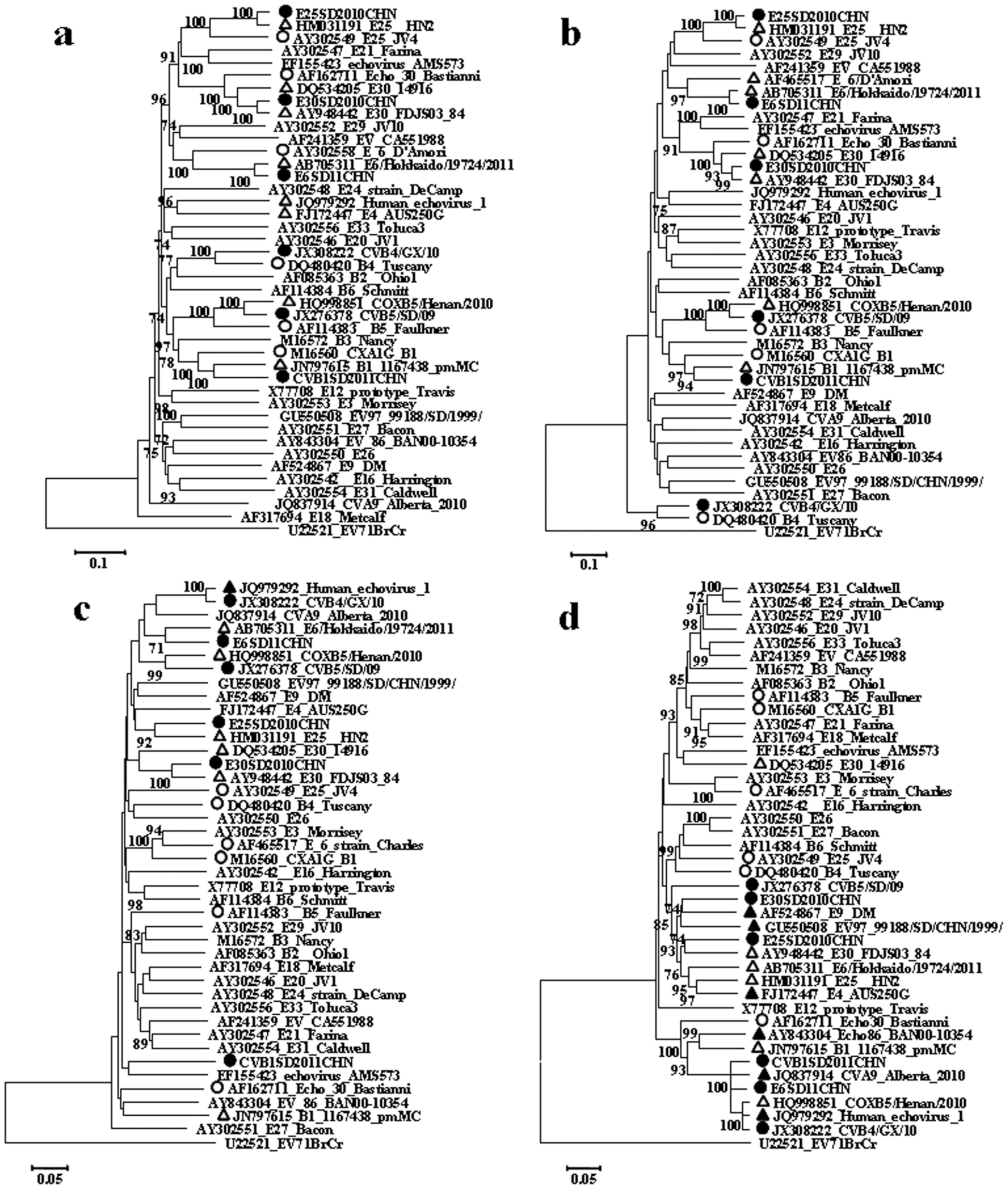
Phylogenetic dendrogram based on comparisons of different genomic regions of the 21 prototype strains and 20 modern strains (including isolates) in HEV-B species using MEGA (version 5.0) and the maximum-likelihood method. (a) P1 region; (b) VP1 region; (c) P2 region; (d) P3 region. Bootstrap values (percentage of 1000 pseudo-replicate data sets) >70% were omitted for clarity. EV71 was used as outgroup in each tree. •Isolates sequenced in this investigation; △closest strains; ○prototype strains; ▴recombination strains.

The genetic relationships and recombination events between newly isolated HEV-B strains and the other HEV-B strains available in GenBank were investigated by constructing neighbor-joining nt phylogenies for the structural protein region (P1 and VP1) (Figure 7a and 7b), and for the regions encoding NSPs (P2 and P3) (Figure 7c and 7d). In the P1 capsid-coding (Figure 7a) and VP1 regions (Figure 7b), E6SD11, B1SD11, CVB4/GX/10, CVB5SD09, E25SD10 and E30SD10 all clustered with their corresponding modern/prototype strains of Echo6/Hokkaido-JPN-2011/E6 DAmori, CVB1-1167438–pmMC/B1-M16560, CVB4-Tuscany, CoxB5-Henan-2010/B5- Faullaner, E25-HN2/EV25-JV4, and E30-FDJS03_84/E30-Bastianni respectively, with strong bootstrap support. Each of the mature proteins (VP1–VP4) derived from the P1 region corresponded well with the pairwise amino acid sequence identities and reconfirmed the serotype of each virus. However, sequences in the P2 and P3 regions were not closely related, showing polytropic relationships in different genomic positions. In the P2 region, five pairs of sequences clustered together (CVB4-JX-10/JQ979292-E1(100%),CVB5SD/COXB5-Henan(99%),E25SD/E25HN2 (92%) and E30SD/FDJS03-84 (100%), E6SD/Echo6-Hokkaido–JPN-2011 (71%)), but not B1SD/B1-1167438–pmMC, which was separate (Figure 7c). In the P3 region, including 3A–D and the 3′region (Figure 7d), six HEV-B serotypes, CVB1SD11, E6SD11,CVA9-Alberta-2010, COXB5/Henan/2010, CVB4/GX/10 and E1-JQ979292 clustered together with 100% bootstrap support. E25SD clustered with FDJS30 and E30SD clustered with EV97 and E9-DM with 74% bootstrap support. The observed differences in the phylogenetic tree topologies between the capsid and non-capsid regions indicated that recombination might have occurred during the evolution of these viruses.

**Figure 8:**
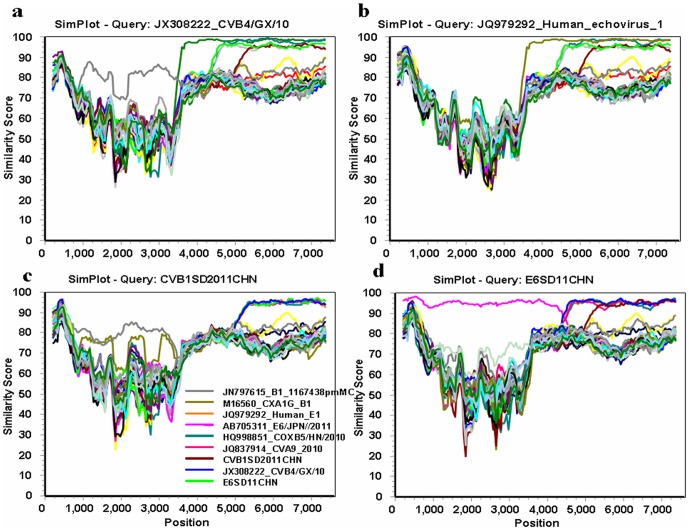
SimPlot analysis based on full-length genomes of the newly isolated HEV-B strains and other HEV-B prototype strains. Each of the B1SD, B4/GX, E6SD and E1-HN sequences was used as the query sequence in each analysis. A sliding window of 400 nt moving in 50-nt steps was used in this analysis. (a) B4/GX; (b) E1-HN; (c) E4SD; (d) B1SD.

**Figure 9:**
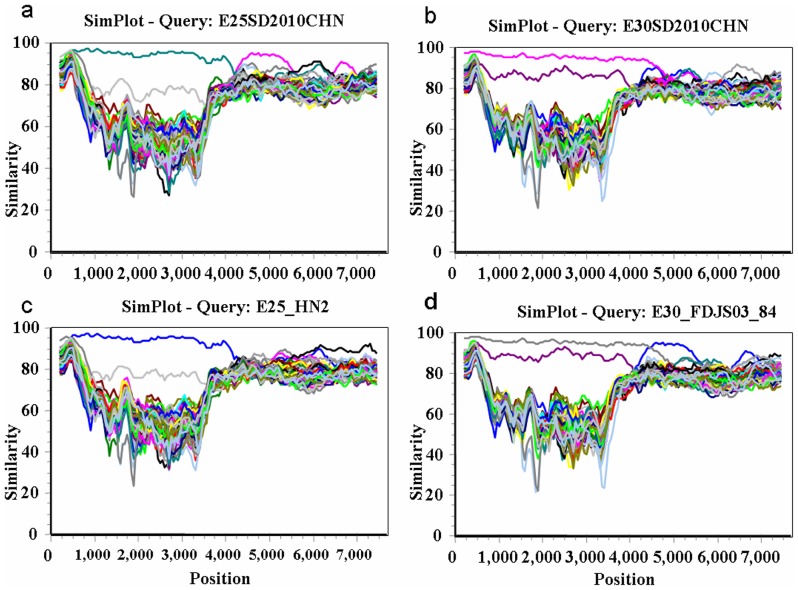
SimPlot analyses based on full-length genomes of the newly isolated HEV-B strains and other HEV-B prototype strains. Each of the E25-HN2 and E30-FDJS03-84 sequences was used as the query sequence in each analysis. A sliding window of 400 nt moving in 50-nt steps was used in this analysis. (a) E25SD; (b) E30SD; (c) E25-HN2; (d) E30-FDJS03-84.

To address the issue of recombination in more detail, the aligned HEV-B complete genome sequences were further analyzed by examining the similarity among sequences in a sliding window of 400 residues, using the SimPlot program. The sequence of each strain was used as the query sequence to generate 40 separate similarity plots. All HEV-B strain 5′NTRs (Figures 8 and 9) were almost equidistant from each other with 85–96% similarity. The VP1 regions of B1SD, E6SD, E25SD and E30SD showed the highest similarities to their prototypes CVB1-conn-5, E6-D′Amori, EV25-JV4, and E30-Bastianni, respectively. CVB5SD, E6SD, E25SD, and E30SD also manifested the highest similarities to their modern strains CVB5-HN, E6-JPN-2011, E25-HN-2, E30FDJ in the 5′ half of the genome (ending around nt 4000–5000 within the P2 region). Interestingly, CVB4/JX/10 was highly similar to at least five other strains, E1-JQ979292, CVB5-HN, E6SD, A9-Alberta, and CVB1 (94–98.7%) throughout most of P2 and P3, in which the similarity plot displayed two obvious cross-points at nts 4667 and 5321 among the five strains (Figure 8a and 8b). The similarity plots for E6SD and CVB1 further confirmed that the CVB1SD, CVB5-HN, CVB4/JX/10, A9-Alberta, E1 and E6SD strains were most similar to each other, with high similarity (94–98.7%) extending from nt 4667 or 5321 to the end of 3D (Figure 8c and 8d). In contrast, E25SD and E30SD exhibited a complicated relationship with the other strains E25-HN, FDJS03, E9-DM and E4-AUS250 in the P3 region. E25SD was closely related to FDJS03 in 2C, 3AB, and part of the 3D region (Figure 9a), whereas it shared the highest similarity with E4AUS250 at the 3C–3D junction region. Similarly, E30SD was related to E25-HN-2 in the 3A–3B region, to EV97 at the 3C–3D junction, and to E9 DM in a portion of 3C with over 95% bootstrap values (Figure 9b). In contrast, the regions of similarity between E25-HN2 and FDJS03 differed from E25SD and E30SD according to the temporal order of strain discovery. E25-HN2 was only related to E4AUS250 in 3D with 90% similarity (Figure 9c), whereas E30-FDJS03 was related to E9 in part of 3C with 85% similarity (Figure 9d). The SimPlot results indicated complex mosaic recombination involving CVB1SD11, E6SD11, CVA9-Alberta-2010, COXB5/Henan/2010, CVB4/GX/10, E1-JQ979292, E25SD, and E30SD.

### Recombination breakpoints according to GARD

The GARD recombination for putative breakpoints within genomes was confirmed using the HKY85 model and the KH test, which demonstrated significant incongruent topologies before and after each breakpoint (Table 1). Firstly, there was evidence of 10 breakpoints among the alignments of CVB1SD11, B1-1167438 pmmC, E6SD11, E6-FJLY2010, CVA9-Alberta-2010, COXB5/Henan, CVB4/GX/10, and E1-JQ979292 using AICc; and an improvement of 5,639.97 points was achieved over the model without recombination. Of those, three breakpoints at nts 3487, 4437 and 5109 also indicated significant topological incongruence using the KH test. Ten breakpoints were also detected in the alignments of E25SD, E25-JV4, E30FDJS-3, E21-Farina, E4-AUS-250G, and B5 2000/CSF/KOR; four topology-flanking positions at nts 924, 3430, 5473, and 6393 were significantly discordant by the KH test. Finally, eight breakpoints were detected in E30SD, E30FDJS03-84, E30-Bastianni, EV97 99188/SD/1999, and E9DM, three of which (nts 4937, 5549 and 6251) were significantly discordant according to the KH test. The topology-flanking points provide evidence for these significant discordant positions as recombination breakpoints. Overall, the GARD analysis results were consistent with the phylogenies and SimPlot results described above, indicating the occurrence of recombination events in these viruses.

**Table 1:** GARD recombination analysis of the HEV-B genome using the HKY85 model.

Alignment	dN	Position#	ΔAIC
aB1/B4/B5/A9/E1/E6	10	72, 91, 667, 1362, 1488, 1911, 1987, 34877***,4437***, 5109***	59.8853
bE25/E30/E4/B5	10	**655** * , 924**, 1227, 1350, 3430***, 4073,5473***,6397***,6875,7045	30.5466
c E30/E9/EV97	10	654, 754, 1915, 3401, 3836, 4736, 4937**, 5549**, 6251**, 7076	19.9917

a B1/B4/B5/A9/E1/E6: Alignment of B1SD11CHN, E6SD11CHN, CVB1-1167438 -pmMC,Echo6/Hokkaido-JPN,CVA9-Alberta-2010,COXB5/Henan/2010,CVB4/GX/10, E1-JQ979292.

b E25/E30/E4/B5: Alignment of E25SD, AY302549.1 E25 JV4, DQ246620.1 E30ZJ, AY302547 E21 Farina, EF155423.1 EAM, FJ172447.1 E4 Aus250G, AY875692.1 B5 2000/CSF/KOR.

c E30/E9/EV97, Alignment of E30SD, E30FDJS03-84, GU550508.1 EV97SD/1999, AF524867.1 E9DM.

d N, number of breakpoints detected.

# Position, breakpoint positions identified via GARD.

* Data in bold font are GARD-identified breakpoints for which topological incongruence was significantly discordant according to the KH test (*p<0.1,**p<0.05, ***p<0.01).

## Discussion

Various enteroviral serotypes are spread globally by temporal and regional factors. The dominant causative agent of HFMD outbreaks in China used to be the C4 genotype of EV71 [22]. We previously investigated the variation in and epidemic characteristics of enteroviruses by performing continuous etiological and epidemiological surveillance in Linyi City, Shandong province, China, in 2008 and 2009, and showed that EV71 was the major pathogen of HFMD [13], [14]. The results of the present study, however, suggest that the pathogenic spectrum changed in 2010 and 2011, with the most prevalent enterovirus serotype being EV71 in 2010 (81%) but CA16 in 2011 (50%). However, the prevalence of HEV-B species isolated from HFMD cases increased from 3% in 2010 to 17.4% in 2011. In all, the isolation of at least 11 HEV serotypes and 16 HEV-B isolates in the 2011 pathogenic spectrum confirmed that enterovirus species exist not as a single genotype, but as a group of independently evolving genome fragments [23], [24]. We tested for the CVB1, CVB4, E6, E25 and E30 serotypes originating from aseptic meningitis outbreaks or other infections in HFMD cases, speculating that HEV-B serotypes may circulate in different clinical diseases in the same region. Our results demonstrated that children younger than 2 years old remained a high-risk population, with an average HEV-positive rate of 68%. A male-to-female ratio of 2.15∶1 was found in severe hospitalized HFMD patients, confirming previous investigations. We also found that HEV-B serotypes, especially E30, tended to be associated with severe cases of HFMD complicated by aseptic encephalitis. This represents an increasing etiology in HFMD outbreaks in eastern China and nearby countries, and should thus be a cause of concern in relation to the prevention and control of HFMD epidemics.

VP1 clustering or genotyping by phylogenetic analysis can discriminate between lineages within a serotype to identify emerging variants or serotypes, and to investigate the molecular epidemiology of enteroviruses. The E25 phylogenetic tree showed that E25 isolates in China in 1998 (Yunnan and Shangdong provinces) were located in cluster B as a result of temporal evolution. However, nearly all E25 isolates found in China in 2000–2010 were located in cluster D3–D6; the only exception being E25-Mongolia AB239934 isolated in Mongolia in 2003, which appeared in the D3 cluster. The E25SD strain and other E25 strains clustered in sublineage D6 (from Shandong, Henan, and Yunnan provinces) and were most closely related to E25-HN isolated from China in 2008, indicating that the E25-HN isolates may be the ancestors of the 2010 Chinese strains. The sequences of the global E30 isolates can be divided into four genetic lineages (A–D) that display at least 15% diversity between clusters, according to VP1 genotypes [25]. E30 strains from China were first detected in Taiwan in 2001 and were classified in subgroup lineage C1. Phylogenetic tree analyses revealed that all E30 strains isolated in China during the last decade occurred in clusters C and D, together with strains Kor08-ECV30 and E30-14125-00 Ukraine 2000 AY896766, which were isolated in Korea (2008) and in the Ukraine (2000), respectively. All E30 stains isolated from Shandong and Jiangsu provinces in 2010–2011 were in Lineage D, along with E30-14125-00 Ukraine 2000, with strong bootstrap support (88%), suggesting that the Ukrainian strain may have been the ancestor of the Shandong and Jiangsu strains in China. Since the outbreak of E30 (subgroup D1) in 2003, the strain was undetected for many years until it reappeared in Shandong province. During this period, a new echovirus sequence emerged that was adapted to the changing environment and challenges. In addition, the phylogenetic tree for E6 was divided into three genetic clusters. The E6SD11CHN strain and other Shandong province E6 strains from environmental sewage in group C6 were most closely related to the E6/Hokkaido strain isolated from Japan in 2011. Moreover, almost all E6 isolates in cluster C6 occurred in 2010 and 2011, and only one, Echo6/Henan127/2008, occurred in 2008. It is possible that the Echo6/Henan127/2008 isolate might be the origin of the 2011 Chinese and Japanese strains. Enterovirus environmental surveillance on sewage from the Shandong Center for Disease Control and Prevention demonstrated that Echo6 was the predominantly active serotype in the cities of Jinan and Linyi, Shandong province, China from 2008–2011 [26], [27]. It would be interesting to determine the transmission pathway of the circulating Echo6 strain in relation to environmental enteroviruses in China, though only one E6SD was detected in the present HFDM cases. The sequences of the global CVB1 isolates can be divided into four genetic lineages (A–D). Phylogenetic tree analyses revealed that CVB1 strains from mainland China were first detected in 1994 and were classified as subgroup lineage D2. The first Shandong CVB1 strain was found in 2000 and tested again after 6 years, and was grouped in sublineages D3 and D4, respectively. Korea CVB1 strains in 2008 belong to subgenogroup D5. Our five CVB1SD strains in 2011 and the recent Zhejiang CVB1 strains in 2009–2010 were classified as a new cluster, sublineage D6. To date, there have been reports of CVB1 activity in the United States during 2007–2008 and in Korea during 2008–2009. The continuing circulation of the CVB1 strain in Korea and China means that we must remain alert to the possibility of additional cases of severe neonatal diseases associated with this strain. All CVB4 strains isolated from China in 2000–2011 were in lineage D. The earlier Chinese strain B4 located in sublineage D1 was isolated in Shandong province in 2000 and 2003. The other CVB4 isolates found in China in 2007–2011, including CVB4SD strains, were all located in a separate cluster D2, and were mainly distributed in Shandong, Shanghai, Guangxi and Shenzhen in southeast China. There have been many reports in recent years concerning the evolution of EV71 and CA16.

To further understand their genetic characters, we sequenced four full-length HEV-B genomes (CVB1SD11, E6SD11, E25SD10 and E30SD10) and aligned them using the ClustalX sequence alignment tool. Based on the updated VP1 sequences in GenBank, the VP1 protein sequences of CVB1SD11, E6SD11, E25SD10 and E30SD10 were 88.9, 93.6, 95.8 and 92.7% identical to those of the corresponding prototype strains, respectively. These are the first complete genomic sequences for recent HEV-B isolates associated with HFMD cases in China. A number of HEV-B serotypes co-circulate thus facilitating spatial and temporal genetic recombination events [18], [28]. Moreover, most circulating HEV-B viruses have incurred multiple recombinations in the NSP region, which commonly results in mosaic enteroviruses [29], [30], [31], [32], [33]. The present study showed that recombination occurred during the 2010-2011 HFMD outbreaks. In the structural protein region (P1), E6-SD11 and CVB1-SD11 were most closely related to their respective homotypic prototype strains, whereas in the NSP region, the epidemic E6-SD2011CHN and CVB1-SD2011CHN strains were highly similar to one another, suggesting genetic rearrangement between the E6SD2011 and CVB1SD2011 strains. Interesting, our SimPlot and bootscaning analyses showed that the CVB1SD11, E6SD11, CVA9-Alberta-2010, COXB5/Henan/2010, CVB4/GX/10 and E1-HN serotypes shared sequences throughout most of the P2 and P3 regions, suggesting multiple recombination events. Of those, COXB5/Henan/2010 (HQ998851) and E1-HN (JQ979292) were isolated in 2010 from Henan province, China, and CVB4/GX/10 (JX308222) was isolated in our laboratory in 2010, from Guangxi, China. Their simultaneous presence in neighboring regions would have provided ample opportunity for co-infection and recombination. In present study, we first report the co-occurrence of intertypic recombination within three diverse species, Coxsackievirus A, Coxsackievirus B and echovirus. E25SD showed high similarity to E25-HN strains in its 5′ half, and shared sequences with E30FDJS03 and E4AUS250 in the P2C–P3D region in a discrete manner. Similarly, several recombination sites were also identified in the E30SD genome, in which the regions of similarity were not contiguous among E30SD, E30FDJ, E25-HN, EV97SD and E9 DM strains. Obviously, recombination between E25SD and E30SD only occurred within echovirus species. Thus identical and different HEV-B serotypes co-circulate and recombine within the same geographical area during outbreaks, and recombination produces new variants with modified pathogenic properties. New variants possess serotype-specific capsid protein sequences, but share non-capsid protein sequences of different origins [34]. Given that the analyzed epidemic CVB1SD11 and E6SD11 strains were derived from recombination events, reconstruction of the ancestral state suggests that E1-JQ979292HN-2010 was the donor of the NSP sequences in the genomes of CVB1SD11, E6SD11, CVA9-Alberta-2010, COXB5/Henan/2010, CVB4/GX/10 and E1-JQ979292. Consequently, co-circulation of the CVB1SD, E6SD, E25SD and E30SD strains isolated from HFMD cases with other echoviruses over 2 years resulted in complex mosaic recombination in Shandong and adjacent regions. Attention should therefore be paid to the possibility of additional cases of severe neonatal diseases caused by E30, E25, E6, CVB1, and their derivatives.

In conclusion, the four HEV-B serotypes, E25SD, E30SD, CVB1SD11 and E6SD11 isolated from HFMD patients in Linyi city differed from strains previously isolated in other continents, forming a novel subgenogroup. Given the prevalence and recombination of these viruses in the HFMD outbreaks, persistent surveillance of HFMD-associated HEV-B pathogens is required to allow the prediction of potential emerging viruses and related disease outbreaks.

## Materials and Methods

### Ethics Statement

This work received approval from the Clinical Ethics Committee of the Institute of Pathogen Biology, Chinese Academy of Medical Sciences and Peking Union Medical College. Written informed consent was obtained from the next of kin, caretakers, or guardians on the behalf of the minors/children participants involved in our study. The ethics committee specifically approved this consent procedure.

#### Samples and virus isolation

Throat swabs were obtained from hospitalized HFMD patients at Linyi People's Hospital (Shandong province, China) during outbreaks in 2010 and 2011. Viral RNA was extracted using an RNeasy Mini kit (Qiagen, Valencia, CA, USA) and stored at −80°C for further use. A total of 233 clinical specimens were subjected to diagnostic reverse transcription-polymerase chain reaction (RT-PCR) generating 350-bp amplicons corresponding to a highly conserved domain in the 5′ non-coding (NCR) of HEV. HEV strains were isolated from positive throat swabs by co-culturing in human rhabdomyosarcoma, Vero and buffalo green monkey cells. To avoid viral mixtures, serially diluted samples were prepared and inoculated into cells in 96-well plates, then the most dilute sample that produced a cytopathogenic effect (CPE) was expanded [35]. Viral mixtures were excluded by sequencing the VP1 and 3D genomic regions of individual clones using the TOPO® TA Cloning® Kit (Invitrogen, Carlsbad, CA, USA) [36].

#### Sequencing

Enterovirus serotypes were confirmed using sensitive semi-nested PCR of VP1 gene sequences with the primer pair AN88-89 [37]. Specific primers for four HEV-B strains, CVB1SD, E6SD, E25SD and E30SD, were designed based on the available GenBank genomic sequences for CVB1-1167438-pmMC (JN797615.1), Echo6-Henan127/2008 (HM185056.1), E25-HN2(HM031191.1) and E30-FDJS03-84 (AY948442). Overlapping genome fragments were amplified using a Qiagen OneStep reverse transcription polymerase chain reaction kit (Qiagen). The PCR products were purified for sequencing using the QIA quick PCR Purification Kit (Qiagen). All strains were sequenced by automated methods using fluorescent dideoxy-chain terminators (Applied Biosystems).

#### Sequence analysis

The amplicons of cDNA sequence fragments were evaluated and assembled manually. The full-length genome sequences of CVB1SD, E6SD, E25SD and E30SD were aligned using Clustal W (http://www.clustal.org/). All sequences were deposited in the GenBank sequence database (18 HEV-B strains accession numbers: KC411818–KC411825, JQ713883–JQ713888, JX976769–JX976773). The resulting alignments were analyzed using SimPlot software version 3.5.1 (http://sray.med.som.jhmi.edu/SCRoftware/simplot/) with a sliding window of 400 nucleotides (nt) moving in steps of 50 nt, with each strain as a query in each run. Phylogenetic trees for 3 distinct genomic regions were constructed with the Molecular Evolutionary Genetics Analysis (MEGA) version 5.0 software package (NJ algorithm and Kimura evolution model) [38], [39]. Recombination analyses were implemented via the Genetic Algorithms for Recombination Detection [32] program in the DataMonkey software package (http://www.datamonkey.org). Multiple breakpoint detection between the non-recombinant and recombinant models was assessed by comparing the corrected Akaike's Information Criterion (AIC_C_) scores. The Kishino-Hasegawa (KH) test was applied to verify whether the adjacent sequence fragments yielded significant topological incongruence. Ancestral-state reconstruction was performed using the neighbor-joining-tree method with DataMonkey software.

## Supporting Information

Figure S1 **Phylogeny of EV71 based on 475 nucleotides of the VP1 gene generated by the neighbor-joining algorithm implemented in MEGA (version 5.0) software using the Kimura 2-parameter substitution model and 1000 bootstrap pseudo-replicates.** •Strains isolated in this investigation. ♦Strains isolated from Shandong. ○Strains isolated from other provinces of China.
(TIF)

Figure S2 **Phylogeny of CA16 based on 475 nucleotides of the VP1 gene generated by the neighbor-joining algorithm implemented in MEGA (version 5.0) software using the Kimura 2-parameter substitution model and 1000 bootstrap pseudo-replicates.** •Strains isolated in this investigation. ♦Strains isolated from Shandong. ○Strains isolated from other provinces of China.
(TIF)
