# Supplementary figures and images for: Epidemics and Frequent Recombination within Species in Outbreaks of Human Enterovirus B-Associated Hand, Foot and Mouth Disease in Shandong China in 2010 and 2011
Source: PLoS One. 2013 Jun 19;8(6):e67157. doi: 10.1371/journal.pone.0067157 (PMC3686723; doi:10.1371/journal.pone.0067157)

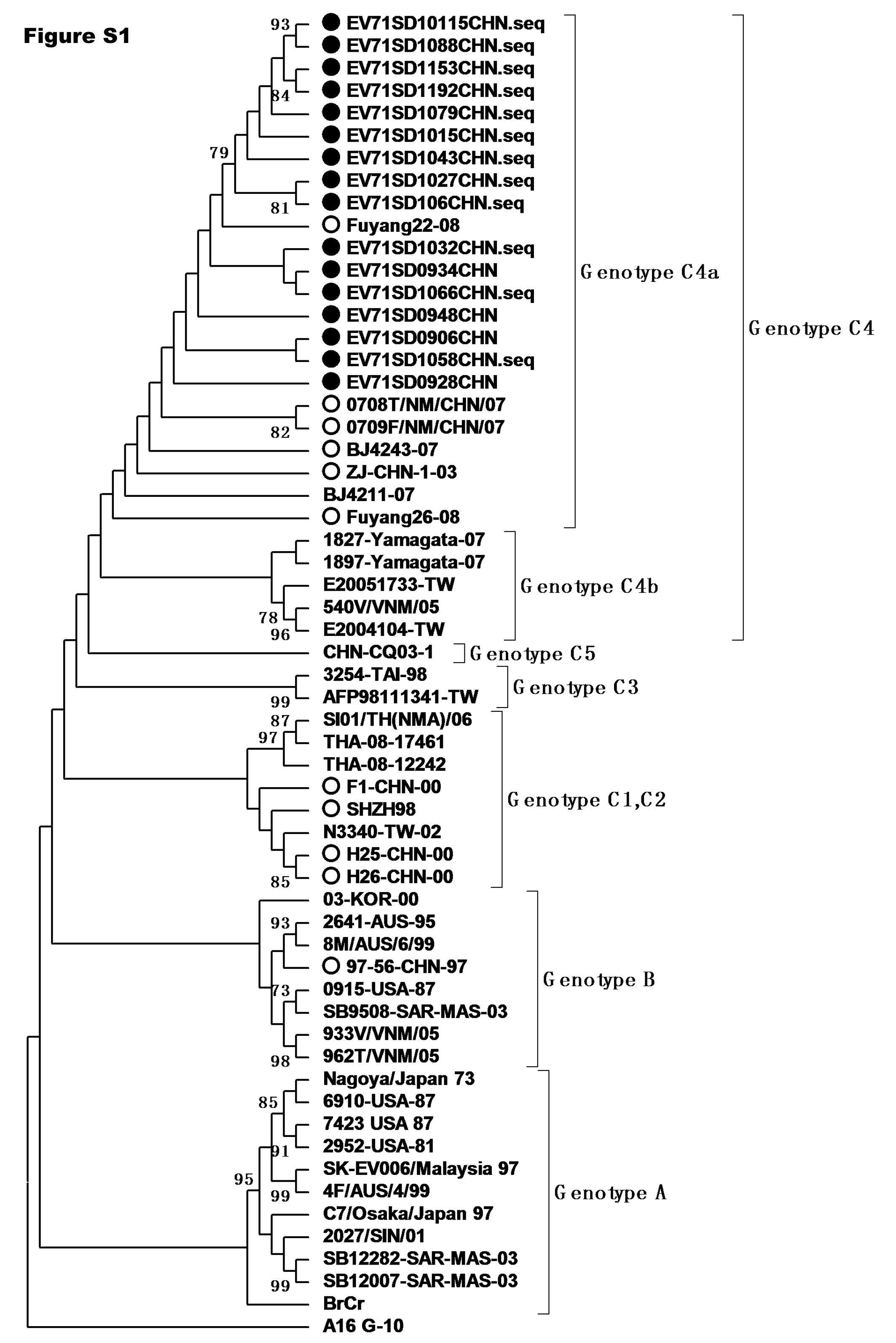

Supplement: Figure S1 — Phylogeny of EV71 based on 475 nucleotides of the VP1 gene generated by the neighbor-joining algorithm implemented in MEGA (version 5.0) software using the Kimura 2-parameter substitution model and 1000 bootstrap pseudo-replicates. •Strains isolated in this investigation. ♦Strains isolated from Shandong. ○Strains isolated from other provinces of China. (TIF) [file pone.0067157.s001.tif]

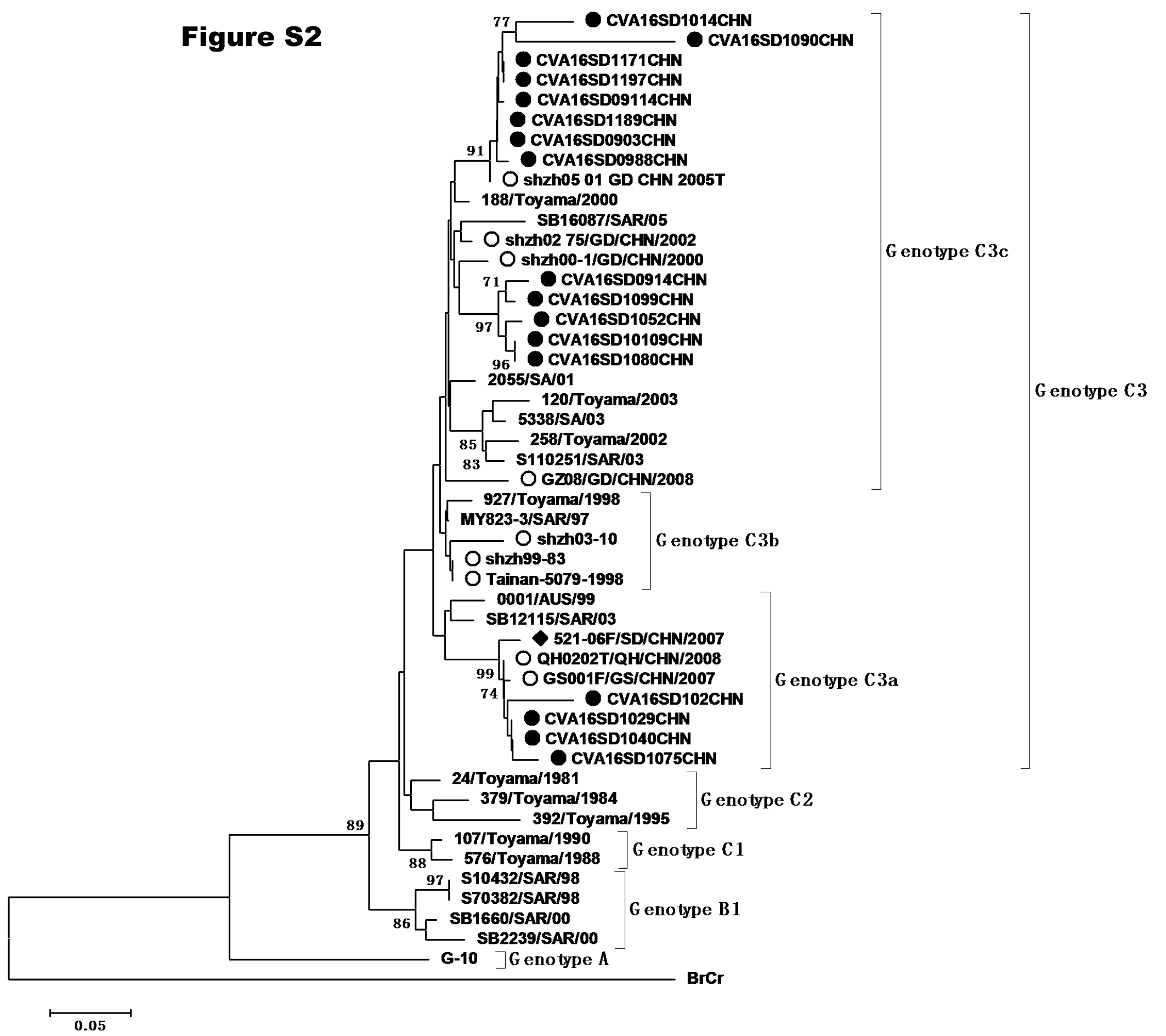

Supplement: Figure S2 — Phylogeny of CA16 based on 475 nucleotides of the VP1 gene generated by the neighbor-joining algorithm implemented in MEGA (version 5.0) software using the Kimura 2-parameter substitution model and 1000 bootstrap pseudo-replicates. •Strains isolated in this investigation. ♦Strains isolated from Shandong. ○Strains isolated from other provinces of China. (TIF) [file pone.0067157.s002.tif]
